# Notes upon Diptheria

**Published:** 1860-03

**Authors:** John H. Hollister


### NOTES UPON DIPTHERIA.

By JOHN H. HOLLISTER, M. D.

In a previous paper I submitted the history of a case of dip theria, in which there was expulsion of false membrane more perfectly developed, and giving a perfect cast of the trachea and bronchial tubes to a greater extent than I have any where noticed in the history of this disease.

The subject was a vigorous lad of 7 years, the inflammation of most decided sthenic character, with remarkably plastic effusion resulting in the formation of false membrane, so firm as to be freely examined, and which still continues to represent the perfect outline of the trachea and larger bronchial tubes. The history of that case was furnished at the request of the Chicago Medical Society, the specimen having been presented for examination before that body.

We seem most to need a careful description of the various pathological appearances and symptoms which occur in the progress of this disease and its *various modifications*, to determine its pathology and appropriate treatment.

A second case of diptheria occurring at the same time and in the same family as the one previously reported, is of especial interest from the fact that in the latter instance there was no formation of false membrane whatever.

The two patients were quite dissimilar in physical development, and as in the first instance there was remarkable fibrilation of the diptheric effusion; so in the latter, there was an entire absence of any fibrous formation whatever, and the extreme phases of diptheria as developed in *sthenic and asthenic* patients could hardly be found in more perfect contrast.

Katie F., aged 5 years, of slight figure and delicate appearance, had suffered for four or five days with what was termed a "croupy cough." When I was called to visit the older brother, (whose case was the subject of my first report) Oct. 4th, 1859, she was deemed so much relieved that my attention was not directed to her.

During my second visit, I noticed her more particularly, and hearing her cough made a careful examination of her throat, and soon convinced myself that she too was suffering from diptheria. The congestion, though intense, was of a passive character; the tonsils were much enlarged, and the whole of the mucous membrane in the posterior part of the mouth of a dark livid color. There was but slight secretion of mucous, the parts were enormously congested, and the labored respiration indicated the same condition of the trachea and bronchial tubes as was so apparent in the fauces. The results of asphyxia began to develope themselves as early as the morning of the second day, and the appearance of the mucous membrane was much changed. From being smooth, nearly dry, and of a dark and almost shining appearance, the parts were now covered with an abundant sanious discharge, which seemed to be discharged from the entire congested surfaces. The application of the sponge was followed by profuse expulsions of the same character from the trachea. The discharge from the nose indicated that the schneiderian membrane was also involved in the difficulty. The aeration of the blood was very imperfectly performed, from the abundance of this secretion in the bronchial tubes. Though the expectoration was profuse, yet no effort on the patient's part afforded other than temporary relief. The livid appearance from capillary congestion early indicated the inevitable result, and she sank with scarcely a struggle in two days and a half from my first attendance.

In this instance there was general vital prostration from the first, and the lowest grade of inflammatory action. The sanious discharge from the over-distended capillaries indicated their entire want of tone, and the effusion was perfectly aplastic in character.

It should be mentioned, that nearly a year previous to the date mentioned, both the children had suffered severely from scarlatina. There had been in each instance severe inflammation of the glands of the neck. After partial recovery, in each case, a purulent abscess pointing outward required to be discharged with the lance. In the first instance there was a perfect recovery from the scarlatina, but in the second the recuperative powers of the little girl never seemed sufficient to restore her to her former health. Her recovery was at best but partial, and being more than usually delicate, she was in every respect a fit subject for the development of asthenic diptheria.

The treatment in this case consisted of Quinine and Muriate tincture of Iron, with gargles of Cayene and Chlorate of Potash, and local applications of Nitrate of Silver in solution, in addition to a nourishing diet.

